# P05-15 Effects on heart rate, physical activity and ambulatory blood pressure from occupational physical activity with and without lifting among farmers in Denmark

**DOI:** 10.1093/eurpub/ckac095.082

**Published:** 2022-08-29

**Authors:** Mette Korshøj, Mathilde Baumann, Michael Hecht Olsen, Ole Steen Mortensen

**Affiliations:** 1 Department of Occupational and Social Medicine, Holbæk Hospital, Holbæk, Denmark; 2 Department of Internal Medicine, Holbæk Hospital, Holbæk, Denmark; 3 Department of Public Health, Section of Social Medicine, University of Copenhagen, Copenhagen, Denmark

**Keywords:** Prevention of cardiovascular disease, Farmers, Hypertension, Occupational medicine, Physical activity and Health paradox, Work Environment

## Abstract

**Background:**

High levels of occupational physical activity associate to increased risk of cardiovascular disease. However, knowledge regarding the acute effects of different components of the occupational physical activity, such as lifting, on risk factors for cardiovascular disease remains uninvestigated during every day work. Thus, the aim was to investigate the acute effects from exposure to occupational physical activity with and without lifting on heart rate, physical activity and ambulatory blood pressure.

**Methods:**

A randomized cross-over study among 18 farming workers in Denmark, all working in the stables of pig- producing farms. Workday measurements of heart rate (Actiheart), physical activity (Axivity placed at front thigh and upper back) and ambulatory blood pressure (Spacelabs 90217, measuring every 20th minute) were collected at a workday with and a workday without occupational lifting. The wash out period between the measurements was 48 hours. Data were processed in the Acti4 software.

**Results:**

During workdays with lifting compared to workdays without lifting we observed higher intensity of occupational physical activity (Δ 6.57% heart rate reserve, 95% CI -1.34 – 14.47), number of steps/workday (Δ 4,965 steps, 95% CI -0.01 – 0.01), standing/walking activities (Δ 83 min/workday, 95% CI 2.49 – 168.97), as well as higher heart rate (Δ 9.10 bpm, 95% CI -4.66 – 22.85) and higher ambulatory blood pressures, both systolic (Δ 3.77 mmHg, 95% CI -2.64 – 10.18) and diastolic (Δ 1.37 mmHg, 95% CI -2.52 – 5.26). The average burden of the occupational lifting were 2,425 kg/workday and amount of lifts/workday were 239 lifts.

**Conclusions:**

This pilot project indicated that occupational lifting are adding strenuousness on top of the general occupational physical activity, and influence blood pressure and heart rate at clinically relevant magnitudes. Disentangling the potential relations between one component of occupational physical activity, such as lifting, and risk for cardiovascular disease is key in the development of initiatives for specific prevention, exposure recommendations and vocational rehabilitation.

